# The Effect of Digested Manure on Biogas Productivity and Microstructure Evolution of Corn Stalks in Anaerobic Cofermentation

**DOI:** 10.1155/2018/5214369

**Published:** 2018-04-23

**Authors:** Zongyan Lv, Lei Feng, Lijie Shao, Wei Kou, Peihan Liu, Peng Gao, Xiaoying Dong, Meiling Yu, Jiuzhang Wang, Dalei Zhang

**Affiliations:** ^1^College of Energy & Environment, Shenyang Aerospace University, Shenyang 110136, China; ^2^Liaoning Institute of Energy Research Co., Ltd, Yingkou 115005, China; ^3^Liaoning Haosheng Biogas Power Generation Co., Ltd, Shenyang 110400, China

## Abstract

The anaerobic fermentation of crop straw and animal wastes is increasingly used for the biogas and green energy generation, as well as reduction of the environmental pollution. The anaerobic cofermentation of corn stalks inoculated by cow dung was found to achieve higher biogas production and cellulose biodegradation. In this study, the effect of mixing corn stalks with cow dung at five different fermentation stages (0, 7, 15, 23, and 31 days of the total fermentation cycle of 60 days) on the further cofermentation process was explored, in order to optimize the corn straw utilization rate and biogas production capacity. In addition, the straw microstructure evolution was investigated by the SEM and XRD methods to identify the optimal conditions for the straw biodegradation process enhancement. The five test groups exhibited nearly identical total biogas productivity values but strongly differed by daily biogas yields (the maximal biogas generation rate being 524.3 ml/d). Based on the degradation characteristics of total solids (TS), volatile solids (VS), and lignocellulose, groups #1 and #3 (0 and 15 days) had the most significant degradation rates of VS (43.73%) and TS (42.07%), respectively, while the largest degradation rates of cellulose (62.70%) and hemicellulose (50.49%) were observed in group #4 (23 days) and group #1 (0 days), respectively. The SEM analysis revealed strong microstructural changes in corn stalks after fermentation manifested by multiple cracks and striations, while the XRD results proved the decrease in peak intensity of cellulose 〈002〉 crystal surface and the reduced crystallinity after cofermentation. The results of this study are assumed to be quite instrumental to the further optimization of the corn stalk anaerobic digestion by inoculation with digested manure for lignocellulose degradation enhancement and biogas productivity improvement.

## 1. Introduction

Large agricultural countries, which produce food and livestock products, have to utilize the by-products, including crop straw and livestock manure. Thus, in China, the annual production of crop straw in 2015 amounted to about 810 million tons, the corn straw share being about 36% or 290 million tons, while the respective livestock manure annual production exceeded 2 billion ton [1]. Crop straw is a multipurpose renewable biological resource, which contains nutrient elements such as C, N, P, K, Ca, Mg, and many organic matters like cellulose, hemicellulose, and protein, but a large part of it is discarded or burned, which not only wastes biomass but also causes a serious environmental pollution. Manure also contains organic matters and nutrient elements, which makes it a good fertilizer, but it is not fully utilized as well. Since inappropriate treatment of crop stalks and livestock manure causes great stress to the environment, their comprehensive and efficient utilization is of great significance for saving bioresources, environmental protection, and agricultural development improvement.

Anaerobic digestion (AD) is one of the renewable energy production technologies, wherein wastes are efficiently treated and the residue (digestate) from the process can be returned to farmland as a biofertilizer [2]. The anaerobic digestion technology can effectively solve the above problems and convert the renewable straw and livestock waste resources into energy substances, such as biogas and industrial ethanol [3–7]. More and more researchers address the anaerobic fermentation [8, 9], and many countries have already launched biogas projects [10, 11]. However, the anaerobic fermentation of pure waste materials exhibited many disadvantages, including nutritional imbalance, acidification, weak buffering capacity, high ammonia nitrogen content, and long chain fatty acid suppression [12, 13], which affect the stability of biogas-generating systems. Therefore, experts have combined various organic compounds to make anaerobic fermentation [14, 15] and revealed that their codigestion can not only avoid the limitation of single raw material fermentation but also improve the utilization efficiency of biomass resources and ensure the joint treatment of various wastes [10, 16–19]. In particular, Jang et al. [20] studied the mixed anaerobic fermentation of wastewater and activated sludge and reported the best organic removal rate and biogas yield when the wastewater and activated sludge ratio was 3 to 4. Zheng et al. [21] found that the anaerobic fermentation of mixed cow dung and switchgrass outperformed that of pure switchgrass by the buffering ability, anaerobic digestion efficiency, and the methane production (which increased by 39%). Codigestion has become an important development trend of anaerobic fermentation technology.

The crop straw structure is complex and includes lignocellulose (lignin, hemicellulose, and cellulose). In turn, the main components of cellulose and hemicellulose are hexose and pentose, respectively, where degradation occurs during the anaerobic fermentation, in contrast to lignin, as reported by Fernandes et al. [22]. A separate fermentation of straw exhibits easy acidification, long fermentation cycle, low degradation efficiency, yield and utilization of straw, and so on [23]. In contrast to straw, livestock dung has a lower carbon-nitrogen ratio and contains numerous nitrogenous substances, including protein, which are decomposed into ammonia nitrogen and exert a buffer effect on the pH drop caused by volatile fatty acids (VFA) [24]. Therefore, the codigestion of animal waste and straw can solve the above problems, improve their utilization rate, enhance the biogas yield, and shorten the fermentation cycle. In recent years, the codigestion of livestock manure and crop straw has attracted attention of international researchers, and some successful achievements were reported. Thus, Gebert and Groengroeft [25] revealed that the addition of 40% of wheat stalks and 100% of rice straw to cow dung increased the daily biogas production by 10.2% and 88.1%, respectively. Liu et al. studied the codigestive performance effect of varied proportions of pig dung and rice straw and reported the optimal ratio, which made the biogas peak appearance acceleration by 11 to 15 days and the maximum yield improvement by 85~265 mL compared to the separate fermentation.

Although the codigestion of crop straw and livestock manure improves the utilization rate of waste/raw materials, numerous nondegraded organic matters (mostly, straw residue) remain in the biogas slurry. This issue was addressed in this study on anaerobic cofermentation of cow dung and corn stalk. The former was fermented separately, and the resulting fermentation broth obtained at five different stages of the fermentation period was mixed with corn stalk to start the codigestion experiment. The comparative analysis of the respective five test groups, including their biogas production, organic matter degradation rate, and microstructural changes, made it possible to identify the optimal mixing conditions for the biogas yield and organic compound utilization efficiency improvement.

## 2. Materials and Methods

### 2.1. Source and Description of Material

The corn stalks and fresh cow dung (FCD) used in the test were taken from dairy farm in Yingkou, China. The stalks were treated by natural air drying and ground into fragments of 1–3 cm length. The physicochemical properties of the materials are listed in Table 1.

### 2.2. Methods

#### 2.2.1. Experimental Setup

The test equipment consisted of anaerobic fermentation tank, thermostat device, and biogas-gathering device. As an anaerobic reactor, 500 ml serum bottle was used, which was sealed with a rubber stopper, and connected by the hose with a flowmeter to the biogas-gathering device. The constant-temperature water bath was used to maintain the fermentation temperature of (37 ± 1)°C.

#### 2.2.2. Experimental Design

**Figure 1:**
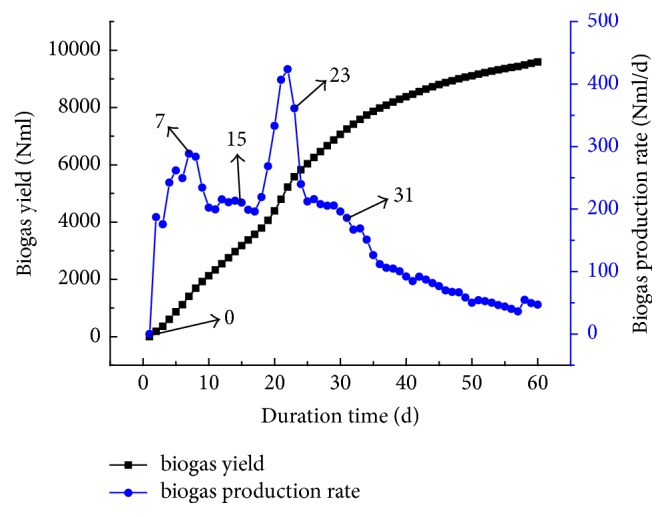
Anaerobic fermentation of fresh cow dung (FCD).

The experimental study involved a mixed anaerobic fermentation of corn straw and cow dung, wherein fresh or partly digested cow dung was added to corn straw, in order to enhance the joint fermentation process. According to the anaerobic fermentation curve of fresh cow dung (FCD) depicted in Figure 1, five terms were selected to add the straw: 0 days/immediate mixing (test group #1), 7 days (test group #2), 15 days (test #3), 23 days (test group #4), and 31 days (test group #5), respectively. These terms corresponded to respective peaks or kink points in the biogas production rate curve (see blue line in Figure 1), while the total fermentation cycle was 60 days.

The 500 ml serum bottle was used as an anaerobic tank, with the active volume of 400 ml. Cow dung and corn straw were mixed in proportion 3 : 1 at different fermentation stages (this is based on mass; strictly controlled mass of corn stalks is 29.17 g for each bottle), and then the completely digested biogas slurry (TS = 5%) was added for dilution (the total proportion of mixed fermentation broth components being equal to 2/3), with the total mass of reactants being equal to 350 g. Each test was repeated twice.

#### 2.2.3. Analytical Methods

The content of total solids (TS) and volatile solids (VS) was determined by the standard APHA methods [26]. Lignin, hemicellulose, and cellulose were determined according to the procedure of Van Soest [27] using a semiautomatic fiber analyzer (ANKOM 200i, Beijing ANKOM Science and Technology Ltd.), with differentiation of neutral detergent fibers (NDF) and acid detergent fibers (ADF). The system of biogas potential testing (AMPTS II bioprocess, Sweden) was used for the determination of biogas yield and production. The X-ray diffraction method was used for the determination of crystallinity (XRD-7000S by Shimadzu Corporation). The ultrahigh resolution field emission scanning electron microscopy (FE-SEM, Nova NanoSEM 450, FEI) was applied to microstructural analysis before and after the fermentation.

## 3. Results and Discussion

### 3.1. Biogas Generation Characteristics

**Figure 2:**
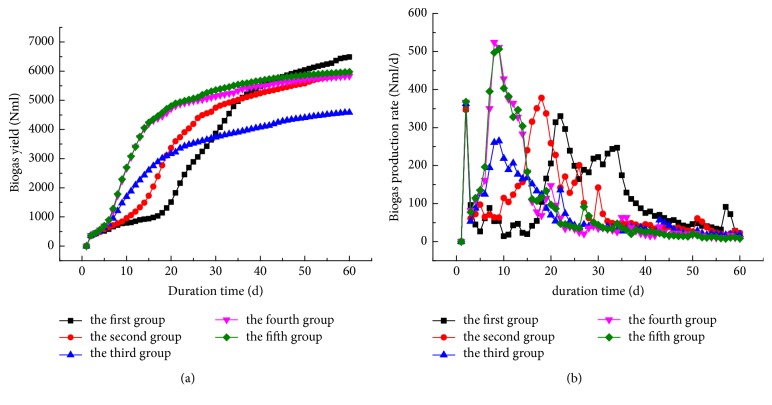
The biogas production of codigestion: accumulative biogas yield (a) and biogas production rate (b).

Biogas production rates and accumulated biogas yields for various test groups of mixed corn stalk and cow dung fermentation filtrate are depicted in Figure 2. As is shown in Figure 2(a), the initial gas accumulation in the decreasing order is exhibited by groups #1, #5, #2, #4, and #3. However, for the total fermentation period of 60 days, the maximum gas yield of 6484.6 ml was observed in group #1 and the lowest one (4583.1 ml) in group #3, which corresponded to 29.3%-difference between these groups. The respective values for the remaining three test groups were nearly identical: 5890.8 ml in group #2, 5829.8 ml in group #4, and 5974.2 ml in group #5.

The respective daily biogas production results are depicted in Figure 2(b), where obvious differences in the five groups can be observed. Each of the five groups has two peaks, while the peak values and positions are different. According to the variation curves of the daily biogas production in each group, longer periods of preliminary anaerobic fermentation of FCD resulted in the earlier appearance of the first peak. Thus, test group #4 had the earliest peak corresponding to day 8, while others were arranged as follows: test group #5 (day 9), test group #3 (day 9), test group #2 (day 18), and test group #1 (day 22). The highest value of the first peak, namely, 524.3 ml/d, was observed in test group #4, the remaining ones being equal to 506.5 ml (test group #5), 264.7 ml/d (test group #3), 378 ml/d (test group #2), and 330.3 ml/d (test group #1), respectively.

The anaerobic fermentation cycle is conventionally subdivided into three phases, namely, start-up phase, stable gas production phase, and decline stage. The start-up phase is critical for ensuring a rapid transfer to the stable gas production stage. Its optimization can shorten the fermentation cycle, thus improving the process-cost and labor-efficiency and saving the material resources [28]. Apparently, the start-up time in different groups significantly differs. Test group #1, which corresponds to codigestion of FCD and corn straw, has the longest time of 18 days. The reason is that there are few anaerobic microorganisms in the FCD, which cause the acidification and hinder the fermentation process after FCD is mixed with corn stalks. At this point, it is essential to monitor the pH value of fermentation reactor, to add the buffer or weak alkaline solution after the pH value decline, in order to provide the neutral pH value, which is more suitable for fermentation. In other groups, there exist some microorganisms in the fermentation broth, which came from the anaerobic-digested cow dung and have a self-adjustment ability of abating the acidification. As seen in Figure 2(b), the other test groups have significantly shorter start-up phase, as compared to that of test group #1. The start-up times of test groups #2 and #3 were ten and six days, respectively. Test groups #4 and #5 exhibited the shortest fermentation start-up time of four days, which was 14 days less than that of test group #1 and implied a strong reduction of the anaerobic fermentation cycle. It is expedient to introduce the notion of effective volume loading rate (EVLR) via the formula

(1) $$\begin{array}{c} EVLR=\frac{Daily\;\;biogas\;\;production}{Fermentation\;\;reactor\;\;volume}. \end{array}$$

Since Figure 2(b) shows that the gas production rate was about 30 ml/d, while the fermentation reactor volume was 400 mL, the respective EVLR amounted to approx. 0.075, where value was too low for the industrial implementation. If the fermentation cycle is readjusted based on the EVLR = 0.1, the fermentation cycles for the five test groups will be 53, 42, 45, 37, and 35 days, respectively. This implies the reduction of the 60-day cycle by 7, 18, 15, 23, and 25 days, respectively, where test groups #4 and #5 have the shortest fermentation cycle. For the new fermentation cycle, the total volume of gas production accounted for 95.71, 90.27, 93.44, 93.03, and 92.75% of groups (#1 to #5), as compared to those in the original cycle, so that the biogas production level of 90% was exceeded in all five groups. This preliminary results provide a reference for the further refinement of the appropriate anaerobic fermentation cycle.

### 3.2. Organics Degradation

**Figure 3:**
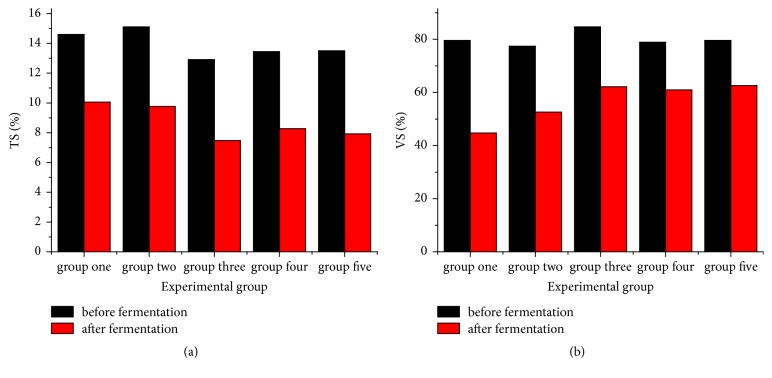
Content variation of TS and VS before and after anaerobic fermentation: (a) TS; (b) VS.

The degradation rate of organic matter is significant for studying the material transformation during fermentation, and it can reflect the anaerobic digestion efficiency of fermentation experiments [29].  Figure 3 shows the total solids (TS) and volatile solids (VS) content variation before and after anaerobic fermentation: the above values are nearly identical in all test groups before anaerobic fermentation but exhibit large differences after fermentation, which reflects their different digestion abilities.  Figure 3(a) depicts the change of TS before and after fermentation. The TS values before the fermentation were 14.6, 15.11, 12.91, 13.44, and 13.51% for test groups #1 to #5, respectively. Here the TS value of group #2 is the highest, and that of test group #3 is the lowest, while the difference in groups #4 and #5 is quite small. The TS values after fermentation were 10.05, 9.76, 7.48, 8.27, and 7.93%, respectively, whereas test groups #1 and #3 had the highest and lowest values, respectively. According to the TS content variation during fermentation, the degradation of TS in the five test groups was 31.16, 35.41, 42.07, 38.47, and 30.57%, which implies that the degradation increased first and then decreased, test group #3 test (adding corn stalks after 15 days of FCD anaerobic fermentation) has the highest degradation rate of TS, and test group #5 (adding corn stalks after 31 days of anaerobic fermentation of FCD) was the lowest, that is, by 27.34% lower than that of test group #3.

Figure 3(b) depicts the VS variation in test groups before and after fermentation. The VS values of the mixed raw materials before fermentation were 79.55, 77.41, 84.7, 78.87, and 79.62%, for the five groups, respectively. The VS content variation of groups is very small, but test group #3 has the highest VS. After fermentation, the VS values in the five groups were 44.76, 52.60, 52.16, 50.96, and 52.58%, respectively. The VS content in test group #1 was the lowest, and nearly identical values were observed in the remaining four groups. According to the VS contents before and after fermentation, the VS removal rates of five groups were 43.73, 32.05, 26.62, 22.71, and 21.4%, respectively. Test group #1 had the highest removal rate, and test group #5 had the lowest one.

Corn straw contains large amounts of lignocellulose, which is composed mainly of cellulose, hemicellulose, and lignin, which are refractory components in straw. Destroying corn straw structure means the disintegration of cell material and the production of humus, while the speed of degradation directly reflects the hydrolysis rate of anaerobic fermentation, which is the most important variation of physical properties in the biological fermentation process.

**Figure 4:**
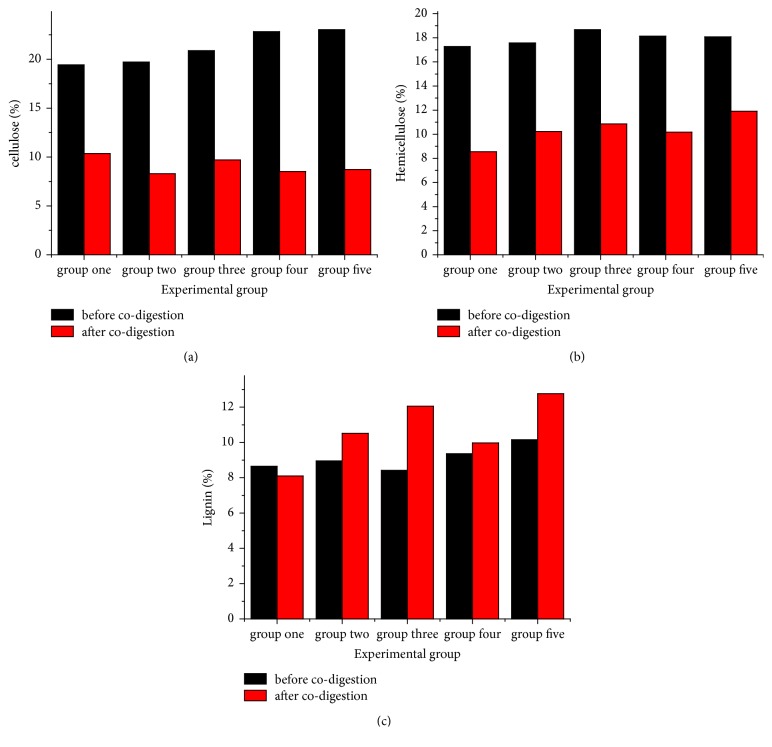
Lignocellulose content variation before and after anaerobic fermentation: (a) cellulose, (b) hemicellulose, and (c) lignin.

The contents of lignocellulose before and after anaerobic fermentation are shown in Figure 4. It can be seen from Figure 4(a) that the cellulose content before the fermentation increased gradually with group number: 19.44, 19.73, 20.89, 22.84, and 23.04%, respectively. For the same content of corn straw, the cellulose content variation during cow dung fermentation has affected the total fiber content. Since the same quantities of cow dung broth were added, the longer fermentation time implied the less dry matter. Therefore, the greater the proportion of straw, the higher the cellulose content. After anaerobic fermentation for 60 days, cellulose content in the five groups reduced by different degrees, which were 10.36, 8.30, 9.71, 8.52, and 8.72%, respectively. Thus, the cellulose content after fermentation was the lowest in test group #2 and the highest in group #1, which implies the cellulose degradation rate of 46.71, 57.93, 53.52, 62.70, and 62.15%, respectively. Since the highest and lowest cellulose degradation rates are observed in groups #4 and #1, respectively, this strongly suggests that cellulose degradation effect of mixed corn straw-cow dung fermentation is the most pronounced when straw is added with 23 days of FCD fermentation, while simultaneous anaerobic fermentation of mixed FCD and corn straw is not beneficial for the cellulose degradation.

Figure 4(b) illustrates the hemicellulose content variation before and after fermentation, where five test groups exhibit similar values before (17.27, 17.56, 18.68, 18.13, and 18.0%) and after anaerobic fermentation (8.55, 10.23, 10.86, 10.17, and 11.90%), respectively. Here test group #1 had the lowest values, and the fifth one had the highest ones. The degradation rates of hemicellulose were 50.49, 41.90, 41.86, 43.91, and 34.22%, respectively, so that the respective parameters of test group #1 (the highest ones) exceeded those of test group #5 (the lowest ones) by 47.55%.

Figure 4(c) depicts the lignin content variation in five groups before and after fermentation. Lignin degradation is extremely hard to achieve in anaerobic fermentation [30, 31]. Lignin content before fermentation was 8.65, 8.95, 8.42, 9.36, and 10.15%, respectively, so that its content in group #5 was the highest. After anaerobic fermentation, the lignin content in five groups was 8.11, 10.52, 12.06, 9.98, and 12.76%, respectively. Once again, the content of lignin in test group #5 was the highest and exceeded that of group #1 (the lowest value) by 57.34%. More detailed analysis of the lignin content variation in five groups before and after the fermentation shows that after fermentation it decreased in test group #1 by 0.54% but increased in the other four groups by 0.57, 3.64, 0.62, and 2.61%, respectively. As compared to the original lignin content before fermentation, the respective variation rates were as follows: −6.21% for group #1; 6.36% for group #2; 43.23% for group #3; 6.62% for group #4; and 25.71% for group #5. Thus, the lignin variation rate in test group #3 was the maximum and its lignin content exhibited the largest increase, while that of test group #1 showed the negative increase.

### 3.3. Scanning Electron Microscopy Analysis

In the process of anaerobic fermentation, the microstructure of corn stalk particles is changed by the action of microorganisms, and damage accumulation and densification of defects can be observed on their surface. The surface morphology of particulate matter reflects the action of particulate matters, microorganisms, and enzymes in the anaerobic fermentation system, eventually, affecting the degradation of organic matters, which constitute corn stalk particles [32, 33].

**Figure 5:**
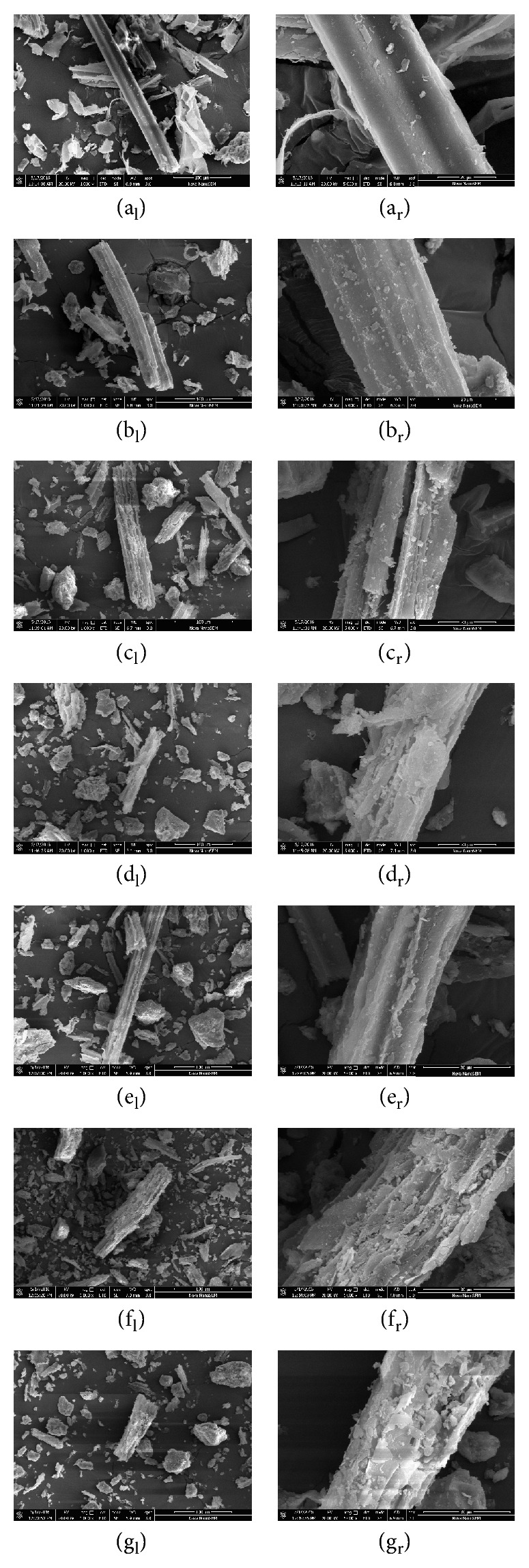
Scanning electron micrographs: magnification of ×1000 (left part) and ×5000 (right part). (a) Pure corn stalk; (b) corn stalk with FCD; (c) test group #1; (d) test group #2; (e) test group #3; (f) test group #4; (g) test group #5.

Figure 5 depicts electron scanning micrographs of the ferment materials, after anaerobic codigestion, where the low-magnification (×1000 or 100 *μ*m) ones are shown on the left side and the high-magnification ones (×5000 or 20 *μ*m) on the right side.

Figure 5(a) depicts the electron scanning micrograph of corn stalk after pulverization. The integral structure of the stalk is yet intact and dense, the surface is smooth, and the density is high. Figure 5(b) presents SEM images of corn stalks mixed with fresh cow dung (FCD). The structure is intact, its surface is more coarse compared with pure stalk, and some streaks are observed, but the density is still high. Thus, both stalks have integral structure, which is not prone to the digestion and degradation for microorganisms, which implies low rate and long-term period of anaerobic digestion and stalk degradation.

Figures 5(c)–5(g) are the electron scanning micrographs of corn stalks mixed with cow dung after fermentation. In 100 *μ*m electron micrographs, the structure of stalks after fermentation, as compared to those before fermentation, is severely damaged and broken up in different degrees, and many ravines appear on their surfaces. In 20 *μ*m electron micrographs, the surfaces of stalks after fermentation look wrinkled, coarse, and uneven. In Figure 5(d), a crevice appears on the straw surface; in Figure 5(e), the surface is partially lifted and slightly cracked; in Figure 5(f), the surface is sunken in and covered by deep crevices; in Figure 5(g), the surface is full of cracks; in Figure 5(h), the surface exhibits cotton-like irregularity and deep cracks. In general, the degradation degree of stalks fermented by the microorganisms is vividly reflected by the electron micrographs. The rougher the surface, the more obvious the crack and the less the density. After analyzing and comparing the scanning electron micrographs of five test groups, the surfaces of stalks in the first three groups (#1 to #3) are found to have more crevices but possess less damaged structures. In the remaining two groups of electron micrographs (#4 and #5), the surface damage was more intense, the degree of biodegradation was more apparent, and fracture of the stalk integral structure was deeper. The results show that in these two groups (#4 and #5) the degradation of cellulose and hemicellulose was more pronounced than that of the first three groups (#1 to #3).

### 3.4. Crystallinity Analysis

In the anaerobic digestion, because of changes in the degree of interbonding of particles, the change of crystallization degree of cellulosic material, and clean size launch tube, the interior of the particle crystallinity is reduced, while the specific surface area of straws is increased, raising their exposure to microorganisms and their secondary metabolites. Each crystalline substance has a specific crystal structure type, while each unit cell can be represented in terms of its lattice parameters. The X-ray diffraction method is used to determine the crystal structure type of particles, for example, as in the recent study of Zheng et al. [28].

**Figure 6:**
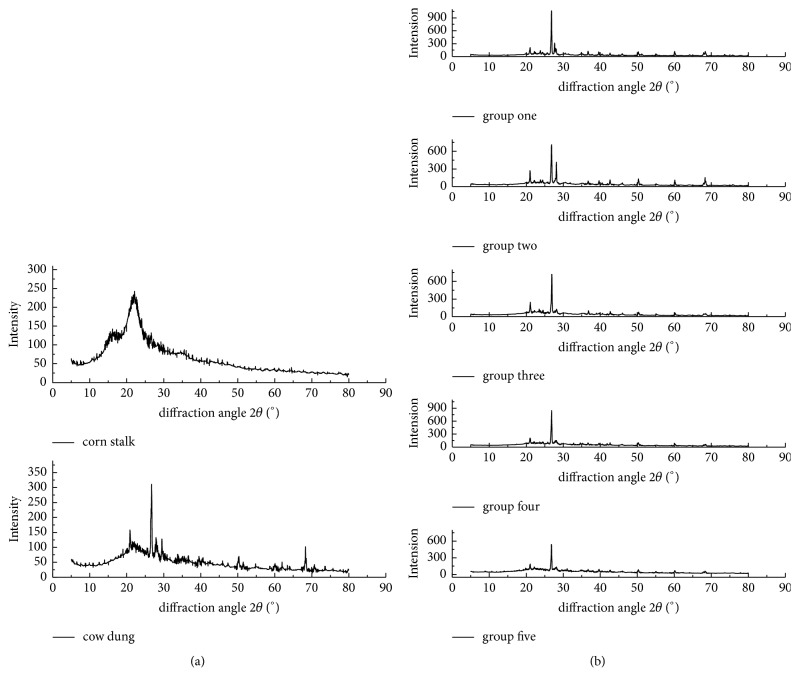
XRD diagram: (a) corn stalk and cow dung and (b) mixed material in five test groups after codigestion.

Figure 6(a) presents the XRD diagrams of corn straw and cow dung before and after fermentation. Evident X-ray diffraction peaks are observed at diffraction angles of about 22°, 24°, and 27°. The above three positions correspond to the diffraction intensity peaks of fiber 〈002〉 crystal surface (C_12_H_22_O_11_), crystalline silica (SiO_2_), and calcium carbonate (CaCO_3_), respectively. Silicate substances and calcium carbonate substances are the essential components of straw cell wall strength, while the composition of cellulose and cell wall changes during the fermentation process. The curves in XRD diagram of corn straw (the upper plot in Figure 6(a)) fluctuate slowly; crests/peaks are not obvious, while burr-type fluctuations are more numerous due to more organic species in corn straw, and there is more interference. The peak appears at about 22°, which corresponds to the diffraction peak of cellulose, while those of silica and calcium carbonate salts are not obvious. In the XRD diagram of cow dung, the crest is obvious, while the burr features are less pronounced, as compared to the corn straw diagram. This may be due to less organic matter and fewer influence factors. The peak intensity of cellulose diffraction decreases at 22°; the peaks are obvious, and the highest one corresponds to that of calcium carbonate.

The XRD diagrams of the five test groups are presented in Figure 6(b). The comparative analysis of graphs in Figures 6(a) and 6(b) reveals that, in the latter ones, the burrs are significantly reduced, the wave lines are relatively smooth, and the peaks are more salient, as compared to the former ones. In all five curves of Figure 6(b), the crest is most pronounced near the diffraction angle of 27°, which corresponds to the diffraction peak position of calcium salts. However, cellulose and silica salts diffraction peaks are much less obvious. It means that the intensity of cellulose diffraction peak decreased significantly, as compared to that of raw materials, while the intensity of the calcium carbonate salts was strongly enhanced. This enhancement can be attributed to the fact that the anaerobic fermentation process consumes a lot of organic matter, thus reducing the relative content of the organic material and increasing the relative content of calcium carbonate and silica salts.

The notion of crystallinity is used to express the proportion of crystalline regions in the fiber: the higher the crystallinity, the larger the crystalline area [34]. The crystallinity of raw and cofermented materials can be assessed via the following formula:

(2) $$\begin{array}{c} Cr=\left(\;\frac{\left(I_{002}-I_{am}\right)}{I_{002}}\;\right)\times \text{100\%}, \end{array}$$

where Cr is the percentage of relative crystallinity, *I*_002_ is the maximum intensity of the 〈002〉 lattice diffraction angle (the diffraction intensity of the crystalline region), and *I*_*am*_ is the scattering intensity of noncrystalline background diffraction with the diffraction angle 2*θ* = 18° [35].

The crystallinity indices of pure corn stalk and FCD were 0.515 and 0.429, respectively, while those of mixed cofermented materials in the five test groups were 0.469, 0.473, 0.357, 0.396, and 0.314, respectively, in which the crystallinity of corn stalk is highest. The crystallinity of the biogas solution after anaerobic fermentation was significantly lower than that of corn stalks by 8.93, 8.16, 30.68, 23.11, and 39.03% for the five test groups, respectively. It is noteworthy that group #5 exhibited the lowest crystallinity of the biogas solution. This strongly indicates that the codigestion can efficiently reduce the crystallinity of cellulose and destroy the crystallization structure, whose effect is the most pronounced in test group #5.

## 4. Conclusions

This study has demonstrated that the mesophilic (i.e., affected by microorganisms growing in moderate temperature range between 25 and 40°C) codigestion of corn stalk mixed with cow dung can improve the biogas production, enhance the degradation efficiency of organic matter, and reduce the anaerobic fermentation cycle. The highest biogas production rate of 524.3 ml/d was observed on day 8 in test group #4 (the codigestion of cow dung after 23 days of anaerobic digestion with corn stalk). Test groups #4 and #5 exhibited better TS and VS removal rates, destroying effect of straw structure fracture, and crystallinity reduction rates.

## Figures and Tables

**Table 1:** Physicochemical properties of materials.

Physicochemical properties	Corn straw	Cow dung
Total solids (TS), %	94.51	10.28
Volatile solids (VS), %	95.21	79.01
Cellulose, %	39.77	20.15
Hemicellulose, %	26.63	18.13
Lignin, %	7.22	10.76
